# The effect of the mobile “blood pressure management application” on hypertension self-management enhancement: a randomized controlled trial

**DOI:** 10.1186/s13063-021-05270-0

**Published:** 2021-06-24

**Authors:** Ali Bozorgi, Hamed Hosseini, Hassan Eftekhar, Reza Majdzadeh, Ali Yoonessi, Ali Ramezankhani, Mehdi Mansouri, Mahnaz Ashoorkhani

**Affiliations:** 1 Tehran Heart Center, Tehran University of Medical Sciences, Shahrivar St., North Kargar St., Tehran, Iran; 2 Center for Research and Training in Skin Diseases and Leprosy, Tehran University of Medical Sciences, Tehran, Iran; 3 Department of Health Education and Promotion, School of Public Health, Tehran University of Medical Sciences, Tehran, Iran; 4 Knowledge Utilization Research Center (KURC), Epidemiology and Biostatistics Department, School of Public Health, Tehran University of Medical Sciences, Tehran, Iran; 5 Department of Neuroscience, School of Advanced Technologies in Medicine, Tehran University of Medical Sciences, Tehran, Iran; 6 Department of Public Health, Shahid Beheshti University of Medical Sciences, Tehran, Iran; 7 Department of Computer Engineering and Information Technology, School of Engineering, Payam Noor University, Tehran, Iran; 8 Department of Health Education and Promotion, School of Public Health, Knowledge Utilization Research Center (KURC), Tehran University of Medical Sciences, Poursina St., Ghods St., Keshavarz Blvd., Tehran, Iran

**Keywords:** Mobile health, mHealth, Hypertension, Medication adherence, Self-management

## Abstract

**Background:**

Self-management of hypertension is of great significance given its increasing incidence and its associated disabilities. In view of the increased use of mobile health in medicine, the present study evaluated the effect of a self-management application on patient adherence to hypertension treatment.

**Methods:**

This clinical trial was performed on 120 hypertensive patients who were provided with a mobile intervention for 8 weeks and followed up until the 24th week. Data on the primary outcome (adherence to treatment) and secondary outcomes (adherence to the DASH diet, regular monitoring of blood pressure, and physical activity) were collected using a questionnaire and a mobile application, respectively. The inter-group change difference over time was analyzed using repeated measures ANOVA (general linear model).

**Results:**

The treatment adherence score increased by an average of 5.9 (95% CI 5.0–6.7) in the intervention group compared to the control group. The scores of “adherence to the low-fat and low-salt diet plans” were 1.7 (95% CI 1.3–2.1) and 1.5 (95% CI 1.2–1.9), respectively. Moreover, moderate physical activity increased to 100.0 min (95% CI 61.7–138.3) per week in the intervention group.

**Conclusion:**

The treatment and control of blood pressure require a multifaceted approach given its complexity and multifactorial nature. Considering the widespread use of smartphones, mHealth interventions can be effective in self-management and better patient adherence to treatments. Our results showed that this application can be used as a successful tool for hypertension self-management in patients attending public hospitals in developing countries.

**Trial registration:**

Iran Randomized Clinical Trial Center IRCT2015111712211N2. Registered on 1 January 2016

## Background

Hypertension has been recognized as a major risk factor for cardiovascular diseases [1]. Approximately 54% of cerebral and 47% of cardiac strokes occur due to hypertension [2]. Over the past 25 years, the number of hypertensive patients and its resultant morbidity and mortality have significantly increased [3]. It has been predicted that by 2025, one out of every three persons will be hypertensive [4]. The hypertension-related morbidity and mortality rate has risen from 97.9 to 106.3 million from 1990 to 2015. Moreover, the disability-adjusted life year (DALYs) has increased from 95.9 to 143 million [3]. Approximately 80% of these problems are seen in low- and middle-income countries [5].

Reducing hypertension and maintaining it in the normal range can prevent its irreparable complications [6]. Hypertension management and treatment is a combination of drug therapy, healthy lifestyle changes—such as regular physical activity, abstinence from alcohol and narcotics abuse, maintaining a healthy weight, and a low-sodium diet—and adherence to treatment [1, 2, 4, 7]. It has been reported that a 10-mmHg decrease in blood pressure can significantly reduce the risk of major cardiovascular disease events [8].

Appropriate adherence to treatment can control hypertension and prevent or delay its complications [4, 7, 9]. Nevertheless, since non-adherence prevails, hypertension is not under desirable control [10, 11].

Given the high prevalence of hypertension across the globe, adherence to treatment has become the focus of many health interventions. The latest in this regard is the utilization of smartphone applications and sensors [12]. Nowadays, smartphone technology is widely used by people everywhere. The penetration rate of mobile phones exceeds 85% across the world population [12]. In Iran too, the penetration rate reached 104.13% from 22% between the years 2006 and 2017 [13]. With a penetration rate of 43.20%, Iran holds the 17th position in the world [14]. The emergence of smartphones has been associated with their utilization in reducing the gaps in access to patients and has been associated with improvements in health services. mHealth is increasingly being used in medical and health fields and for self-management of various health conditions [15].

Many studies have investigated the effectiveness of utilizing smartphone applications, and most of them have reported desirable effectiveness [16–18]. mHealth technologies have experienced rapid and extensive geographical development and have been used across multiple countries due to their appeal, popularity, low cost, and outreach beyond spatial and temporal constraints. Given their low cost, developing countries have especially used the smartphone’s capabilities to educate patients [19].

We used the Predisposing, Reinforcing, and Enabling Constructs in Educational Diagnosis and Evaluation (PRECEDE) conceptual framework to design the educational package of this study (in the form of a smartphone application). The PRECEDE model is a health promotion model that has been recognized as an efficient means of designing and implementing educational interventions [20]. This clinical trial was conducted to evaluate the effectiveness of the Blood Pressure Management Application (BPMAP) application in promoting adherence to pharmacological and non-pharmacological treatments in patients with primary hypertension in Iran among those attending academic medical centers.

## Methods

### Study design

This was a randomized, controlled clinical trial (IRCT2015111712211N2) with two parallel arms designed to demonstrate the effect of BPMAP (Supplementary file 1) on self-management of hypertensive patients in adherence to the treatment and control of hypertension risk factors [15]. This article was written using the CONSORT 2010 checklist (See Additional file 1).

### Study setting

The “Tehran Heart Center” is a tertiary referral cardiovascular subspecialty medical center affiliated to “Tehran University of Medical Sciences” in Iran. Each year, the center has about 113,000 outpatients and 24,000 admissions and performs 4000 open heart surgeries [21].

### Participants

The participants included patients with primary hypertension diagnosed and confirmed by their physician. The inclusion criteria were 30–60-year-olds, being literate (of at least primary education), being treated for hypertension (ICD code I10) for at least 1 year, possession of and the ability to use a smartphone, willingness to participate in the study, and residence in the study region throughout the study period. The exclusion criteria were complications of hypertension such as myocardial infarction (I22) and stroke (I64), other cardiovascular diseases (I51.6), diabetes (E10–E14), and physical disability (Z73.6).

### Interventions

Participants in the intervention group received a mobile application-based educational-supportive intervention, along with the routine treatment. The content and features of the application were prepared based on the educational needs of hypertensive patients [22]. The application had the following features: (a) recording blood pressure and receiving feedback of the recorded blood pressure; (b) saving the recorded blood pressure levels and plotting them in a chart; (c) reminding the time of drug consumption, visit date, and blood pressure measurement; (d) healthy diet (DASH and low-salt diet) and weight loss plans; (e) knowledge-based information on the nature, control, and treatment of the disease; (f) motivational and supportive programs for smoking cessation; (g) sending notifications or informing one of the patient’s family members of critical blood pressure levels; (h) sending general motivational messages and reminders about adherence to treatment to all patients and specific individual messages based on patient characteristics; and (i) saving the recorded information by users on the portal for physicians and researchers.

Participants in the comparison arm received the usual care, including taking their medical history and undergoing physical examinations, such as measurement of blood pressure and weight, laboratory tests and paraclinical services tailored to the individuals’ conditions, and drug treatments according to the JNC8 [23] recommendations.

### Outcomes (primary and secondary)

Adherence to antihypertensive medication was considered as the primary outcome, assessed with the 14-item Hill-Bone Scale (range 14–70) [24]. The secondary outcomes included clinical and behavioral outcomes, such as adherence to the DASH diet, reduced sodium and fat intake, regular blood pressure monitoring, physical activity, and predisposing, enabling, and reinforcing factors in adherence to treatment. Clinical outcomes were measured by a physician, and other outcomes were measured by a questionnaire. The questionnaire was completed by a trained researcher.

#### Adherence to the DASH diet and sodium reduction

The intake of salt, fat, low-fat dairy products, fruits, and vegetables over the past week was evaluated.

#### Regular blood pressure monitoring

The frequency of blood pressure in the application was recorded in the study period.

#### Physical activity

The degree of change in the frequency and time allocated to physical activity was evaluated in two categories of moderate and severe physical activity.

#### Predisposing, enabling, and reinforcing factors in adherence to treatment

The effect of these factors was evaluated based on the questionnaire items.

### Sample size

We calculated that a total of 120 people (60 in each group) would be needed to achieve a power of 80 (1−*β*) to detect at least a 5-score (SD = 10) difference on the Hill-Bone Compliance to High Blood Pressure Scale [24] using the two-sided *T* test at the significance level of 5% (*α*).

### Randomization

After eligibility screening, using random sequence—which was generated by online randomization (www.sealedenvelope.com) in advance—we assigned the eligible subjects to two groups. The random sequence was generated using permuted block randomization with a block size of four and a 1:1 allocation ratio.

### Implementation

**Fig. 1:**
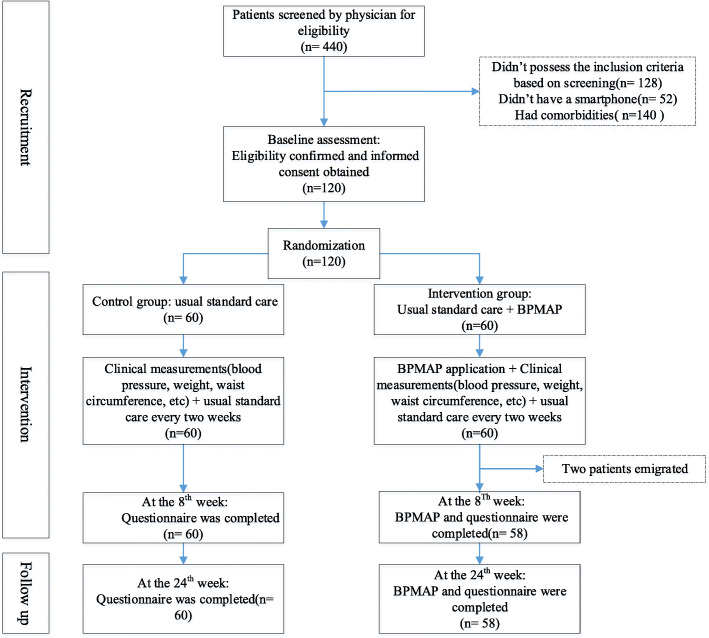
Trial flow diagram. The procedure of the study evaluating the impact of the Blood Pressure Management Application (BPMAP)

Individuals were given 1 week to review and consult with the family to read and sign the informed consent form. After the participants signed the informed consent, the initial assessment questionnaire was completed by a trained interviewer (Fig. 1). The application was installed on the mobile phones of the intervention group, and instructions were provided to the patients individually.

Blood pressure was measured by the “WELCH ALLYN Tycos Jewel Movement Sphygmomanometer” at the clinic. The patients’ sphygmomanometers were calibrated at the time of application installation to ensure correct measurement of blood pressure at home.

### Follow-up assessments

Participants in both groups had an initial visit and six follow-up visits. The first 5 visits were performed at 2-week intervals, and the last one was done 24 weeks after the first visit (Table 1). In both groups, clinical evaluation and questionnaire completion were done at baseline, 8 weeks after receiving the application, and at the 24th week.

**Table 1:** Steps taken in each visit in both groups

	Study period						
	Enrollment	Allocation	Post-allocation				Close-out
Week	0 ± 2 days	0 ± 2 days	2 ± 2 days	4 ± 2 days	6 ± 2 days	8 ± 2 days	24 ± 2 days
Visit	1st visit	2nd visit	3rd visit	4th visit	5th visit	6th visit	7th visit
Assessment of inclusion criteria and sampling	×						
Attainment of informed consent	×						
Baseline assessment	×						
Formation of two groups: intervention and control	×						
The intervention		×	×	×	×	×	×
Measuring adherence to treatment	×					×	×
Measuring adherence to the diet	×					×	×
Evaluating the predisposing, enabling, and reinforcing factors of adherence	×					×	×
Measurement of systolic and diastolic BP, waist circumference, and BMI	×	×	×	×	×	×	×
Evaluating the regular monitoring of BP by the mobile app	×	×	×	×	×	×	×
Evaluating the usability of the software						×	×
Evaluating the level of satisfaction of the software/application						×	×

### Data collection methods

The primary outcome was measured by the Hill-Bone Scale, and secondary outcomes were measured by a mercury sphygmomanometer and the researcher-made questionnaire.

The questionnaire contained patients’ demographic information, treatment status, and some health-related behaviors as the baseline assessment. A number of the Predisposing, Reinforcing, and Enabling Constructs in Educational Diagnosis and Evaluation (PRECEDE) model constructs (predisposing, enabling, and reinforcing factors); the World Health Organization STEPS questionnaire for monitoring chronic risk factors [25]; the Hill-Bone Compliance Scale; and the Global Physical Activity Questionnaire (GPAQ) guide were used in its design [26]. It contained 68 questions for assessing 6 domains. The domains and the number of questions in each are as follows: (a) physical and biochemical measurements (9); (b) assessment of disease history (7); (c) assessment of health behaviors—such as smoking (4), nutritional diet (5), and physical activity (6); (d) predisposing (18), enabling (2), and reinforcing factors (3) of adherence; and (e) Hill-Bone adherence to treatment questionnaire (14). The questions in domain “c” have been taken from the STEPS and the GPAQ questionnaires.

### Data management

The patients’ data were obtained from the self-report questionnaire and the application. Whenever the users would go online, their activity in the application would be stored on the portal (Supplementary file 2: Table 1). The final databank was prepared in SPSS 20.0. A double entry was made to ensure that the data was entered correctly. In the event of any discrepancy, the information entered was verified with the initial data.

### Statistical methods

The baseline characteristics of patients in the two treatment groups were reported using the mean (standard deviation) for quantitative variables (age, cholesterol, blood pressure, weight, adherence parameters) and frequency (frequency percentage) for qualitative variables (sex, marital status, education, frequency of BP measurement).

To analyze the effectiveness of the main variable outcomes, we used repeated measurement analysis of covariance (general linear model) to compare the mean change in variables of interest adjusted to the baseline score at a confidence interval of 95%. We set the intervention group as the fixed and the times of measurement as the repeated factors. All analyses were conducted using SPSS (version 20.0).

## Results

This study was conducted from September 2016 to August 2017 on 120 people. Two participants in the intervention group were lost to follow-up due to emigration and irregular attendance during periodic visits. Males consisted of 56.9% and 60% of the participants in the intervention and comparison groups, respectively. The mean hypertension duration was about 7 years in the intervention group and about 8 years in the comparison group. The mean arterial pressure (MAP) was 108 ± 13.5 in the intervention group and 115 ± 14.3 in the comparison group. The mean medication adherence score was 58.5 ± 7.4 in the intervention group and 59 ± 5.0 in the comparison group (Table 2).

**Table 2:** Baseline characteristics of the study population

	Characteristics	Intervention group (60)	Control group (60)
**Demographic**	Age (years, mean ± SD)*	52.0 ± 8.1	51.6 ± 9.4
	Sex (male)	35 (58/3)	36 (60)
	Marital status		
	• Alone, *N* (%)	3 (5.0)	6 (10)
	• Living with family, *N* (%)	57 (95.0)	54 (90)
	Educational status, *N* (%)		
	• Under diploma	8 (13.4)	7 (11.7)
	• Diploma	33 (56.7)	26 (43.3)
	• Bachelor	12 (20.0)	20 (33.3)
	• Master and above	6 (10.0)	7 (11.7)
**History**	Duration of disease (years, mean ± SD)	7.3 ± 6.2	8.3 ± 6.3
	Duration of medication intake (years, mean ± SD)	5.7 ± 5.3	6.4 ± 5.8
	Number of visits per year for hypertension (mean ± SD)	1.8 ± 2.3	1.9 ± 1.5
	Frequency of home blood pressure measurements, *N* (%)		
	• A few times a week	11 (18.3)	8 (13.3)
	• Once a week	7 (11.7)	10 (16.7)
	• Once a month	2 (3.3)	9 (15.0)
	• Whenever I feel I am not good	31 (51.7)	29 (48.3)
	• Never	9 (15.0)	4 (6.7)
	Cholesterol (mg/dl, mean ± SD)	179.8 ± 38.6	196.7 ± 32.0
	Triglyceride (mg/dl, mean ± SD)	157.4 ± 57.0	163/0 ± 44.4
	LDL (mg/dl, mean ± SD)	107.45 ± 23.78	105.6 ± 28.6
	HDL (mg/dl, mean ± SD)	47.08 ± 12.08	51.9 ± 15.4
	FBS (mg/dl, mean ± SD)	97.03 ± 17.2	96.8 ± 14.5
	MAP* (mmHg)	108.0 ± 13.5	114.9 ± 14.3
	BMI (kg/m^2^, mean ± SD)	29.7 ± 3.4	28.5 ± 3.6
**Characteristics**	Adherence to medication/Hill-Bone checklist (mean ± SD) 3*	58.3 ± 7.8	59.1 ± 5.0
	Adherence to medication/self-assessment (mean ± SD) 2*	15.37 ± 4.6	17.0 ± 3.1
	Adherence to a low-fat diet (mean ± SD) 2*	12.6 ± 5.1	15.8 ± 3.3
	Adherence to a low-salt diet (mean ± SD) 2*	14.1 ± 5.7	15.8 ± 3.4
	Vigorous physical activity (mean ± SD) 4*	170.0 ± 87.4	162.0 ± 100/8
	Moderate physical activity (mean ± SD) 5*	168.8 ± 130.8	113.0 ± 72.9
	Knowledge (mean ± SD) 6*	2.8 ± 2.4	2.1 ± 1.2
	Attitude (mean ± SD) 7*	22.9 ± 5.6	23.8 ± 4.5
	Self-efficacy (mean ± SD) 2*	16.2 ± 2.0	14.7 ± 2.3

1* (range 65–30), 2* (range 1–20), 3* (range 14–70), 4* (range 0–150 min/week), 5* (range 0–300 min/week), 6* (range 1–7), 7* (range 1–35), *$\left(\mathrm{Mean}\ \mathrm{arterial}\ \mathrm{pressure}\right)\ \mathrm{MAP}=\frac{\mathrm{SBP}+2\ \left(\mathrm{DBP}\right)}{3}$

Based on the Hill-Bone Scale, the mean adherence to medication was 65.1 (95% CI 65.04–65.23) in the intervention group and 59.7 (95% CI 59.60–20.36) in the control group, indicating an improvement of 5.9 points (95% CI 5.03–6.69) in the intervention group (Table 3).

**Table 3:** Outcome variables measured at baseline, at the 8th week, and at the 24th week as the final visit, showing changes between visits and group differences

Outcomes	Groups	Baseline assessment, mean ± SD	8th week, mean ± SD	24th week, mean ± SD	Mean change over time (95%CI)
Adherence to medication					
According to the Hill-Bone checklist	Intervention group	58.5 ± 7.42	65.1 ± 2.44	66.1 ± 2.00	5.9 (5.0–6.7)
	Control group	59.1 ± 5.07	59.7 ± 3.69	59.9 ± 3.51	
According to the self-assessment	Intervention group	15.3 ± 4.64	19.5 ± 0.86	19.7 ± 0.71	1.9 (1.5–2.3)
	Control group	17.0 ± 3.14	18.4 ± 1.91	18.0 ± 1.50	
Healthy diet					
Adherence to low-salt diet*	Intervention group	14.3 ± 5.7	18.40 ± 1.18	18.59 ± 0.91	1.5 (1.2–1.9)
	Control group	15.8 ± 3.4	17.30 ± 1.87	17.12 ± 1.41	
Adherence to low-fat diet*	Intervention group	16.0 ± 3.0	17.86 ± 1.06	18.21 ± 0.78	1.7 (1.3–2.1)
	Control group	15.8 ± 3.3	17.13 ± 1.78	16.66 ± 1.72	
Weight loss					
BMI (kg/m^2^)	Intervention group	29.7 ± 3.4	29.3 ± 3.5	28.6 ± 3.2	1.2 (0.77–3.2)
	Control group	28.5 ± 3.6	30.2 ± 15.5	28.4 ± 3.7	
Blood pressure					
MAP (mmHg)	Intervention group	108.1 ± 13.5	95.5 ± 3.66	94.8 ± 3.42	3.4 (1.6–5.2)
	Control group	114.9 ± 14.30	98.9 ± 6.84	100.1 ± 7.20	
Physical activity					
Vigorous physical activity 2*	Intervention group	162.2 ± 83.2	195.0 ± 79.9	173.3 ± 68.6	97.3 (41.1–153.1)
	Control group	162.0 ± 100.8	140.20 ± 69.3	56.7 ± 18.9	
Moderate physical activity 3*	Intervention group	169.4 ± 132.8	260.9 ± 115.9	247.3 ± 96.29	100.0 (61.7–138.3)
	Control group	113.0 ± 72.9	154.6 ± 135.47	102.7 ± 53.33	
Predisposing factors					
Knowledge 4*	Intervention group	2.7 ± 2.3	6.4 ± 1.2	6.4 ± 1.2	2.9 (1.6–4.2)
	Control group	2.1 ± 1.2	2.6 ± 1.2	3.1 ± 1.4	
Attitude 5*	Intervention group	23.0 ± 5.43	26.4 ± 2.82	24.55 ± 4.97	2.3 (1.2–3.4)
	Control group	23.8 ± 4.47	24.13 ± 3.45	22.68 ± 4.48	
Self-efficacy*	Intervention group	16.18 ± 2.03	16.9 ± 1.42	16.9 ± 1.41	1.7 (1.3–2.2)
	Control group	14.75 ± 2.30	14.5 ± 1.76	14.6 ± 1.58	

* (range 1–20), 2* (0–150 min/week), 3* (0–300 min/week), 4* (range 1–7), 5* (range 1–35)

Adherence to the DASH diet was examined with 5 items. Three items were related to dairy, fruit, and vegetable consumption. The findings indicated an increase in dairy, fruit, and vegetable consumption in the intervention group compared to the control group. In addition, subjects in the intervention group adhered to low-fat and low-salt diet plans better than the comparison group. Subsequently, adherence to low-fat and low-salt diet plans increased by 1.7 points (95% CI 1.30–2.10) and 1.5 points (95% CI 1.16–1.90), respectively (for more results, see Supplementary file 2: Table 2).

The MAP decreased over time by an average of 3.4 mmHg (95% CI 1.6–5.2) in the intervention group compared to the control group.

To monitor blood pressure, the recorded blood pressure measurements of the intervention group were entered in the relevant section of the software. A recording frequency of 30 times was expected in the intervention group. Recordings of more than 25 times were considered desirable. The recordings of 84.7% of the application users (50 people) were appropriate. (further data on the users’ activity in the app are provided in Supplementary file 2: Table 2).

The estimated mean of moderate physical activity over the study period was 247.3 (95% CI 223.7–267.5) in the intervention group and 102.7 (95% CI 100.4–176.8) in the control group, indicating an increase of 100.0 min per week (95% CI 61.7–138.3) in the intervention group.

The predisposing factors of adherence to treatment (knowledge, attitude, and self-efficacy) in the intervention group led to an improvement of 2.9 points (95% CI 1.6–4.2) in knowledge, 2.3 points (95% CI 1.2–3.4) in attitude, and 1.7 points (95% CI 1.3–2.2) in self-efficacy among the patients.

The rate of satisfaction of the application was measured on a scale of 1–20 in each visit. The users’ mean satisfaction rate was 18.41 (min 16, max 20). Also, usability was examined by a questionnaire at the end of the study, the results of which are presented in Supplementary file 2: Table 3.

## Discussion

Adherence to treatment in chronic diseases is of paramount importance, which is estimated at 50–60%. Currently, mHealth is increasingly applied to address this problem [22]. The results of this study showed that the use of BPMAP by hypertensive patients resulted in an increase of 5.86 points in patient adherence to medications.

Evidence suggests that 65% of mHealth studies have increased adherence to drug therapy and in some cases have been able to increase patients’ self-efficacy and self-care by sending encouraging messages [27, 28]. However, most of these studies have used text messages and notifications only [28, 29]. It seems that the significant effects of improving adherence to treatment, presented in this study, were due to paying attention to its different aspects and its holistic view, such as, access to treatment knowledge, drug reminders, sending knowledge-based and single-sentence encouraging messages, recording of blood pressure measurements at home, and getting feedback about the recorded numbers.

In BPMAP users, the number of vegetables, fruit, and dairy portions increased, and the consumption of salt and fat decreased. A study was conducted to improve nutritional status using desktop computers, the results of which indicated an increase in the consumption of vegetables and cereal grains [30]. The use of technology to improve nutritional status appears to be effective. Although these studies have not examined the effect of evidence in the long run, it seems that the use of new technologies along with medical treatment is useful.

Blood pressure monitoring at home is increasingly accepted and used by patients and has desirable and helpful impacts on clinical interventions. In the present study, the blood pressure measurement reminder and increased knowledge of the disease have led to regular monitoring of blood pressure at home. About 84.5% of BPMAP users measured and recorded their blood pressure more than 25 times over a period of 2 months. Measurement of blood pressure at home keeps individuals informed of their blood pressure status and promotes adherence to treatment, which can lead to optimal blood pressure control [31].

Regular physical activity is an effective factor in controlling hypertension and improving one’s health. In addition to knowledge about the importance of regular physical activity on appropriate blood pressure control, encouraging messages were sent via the application at 3-day intervals, which increased the average physical activity of BPMAP users by 100 min per week. A similar finding was found in studies conducted in the UK [32] and Taiwan [33]. Contradictory results were also reported on the ineffectiveness of encouraging messages on increased physical activity in a study by Nguyen et al. in the USA [34]. The difference in the effectiveness of encouraging messages can be attributed to the quality, relevance, and format of the messages sent. Accordingly, it is essential to pay attention to the indicators of effective messages when designing them.

Although pharmacological treatment is as important as non-pharmacological therapies in controlling blood pressure, in clinical settings, especially in non-private healthcare systems in which many visits are made, non-pharmacological recommendations are not sufficiently addressed. In these conditions, the use of other methods, such as providing enough and necessary information to patients can improve the level of knowledge, attitude, self-efficacy, and adherence to treatment. A systematic review by Marshall et al. in 16 countries revealed that the lack of awareness of the causes and symptoms of hypertension, fear of the drugs’ side effects, and their dependency are the major causes of treatment discontinuation and lack of adherence to treatment [35, 36]. In this study, BPMAP provided the users with the necessary knowledge which had been identified in pre-intervention studies. Raising public awareness about hypertension and the necessary measures of self-management can help improve individuals’ adherence to pharmacological and non-pharmacological treatments. Therefore, the use of technologies such as mobile applications is recommended in these cases.

In spite of mHealth’s appeal, popularity, low cost, and outreach beyond spatial and temporal constraints, our study had its limitations, which were as follows: the self-report nature of the questionnaire and the need to register the measurements in the application, and the necessity of owning an Android smartphone. Furthermore, our study was not blinded. If our consultant physicians and participants had positive attitudes toward the effects of the application on adherence, they may have been more inclined to detect or express positive results.

## Conclusion

Hypertension is a multifactorial disease that requires a multifaceted approach if it is to be properly controlled. In order to achieve this multifaceted approach, utilizing smartphone capabilities can be a good alternative to drug therapy alone, given their low costs and high penetration. In addition to regular consumption of drugs, patients also need to fully adhere to pharmacological and non-pharmacological recommendations. The present study showed that the proper use of BPMAP is effective in raising the level of knowledge, controlling the factors influencing adherence to treatment, and having access to family support. Given the participants of this study were visiting a non-private center in Iran, the authors believe that this approach could be a successful tool for self-management of hypertension in similar settings in many developing countries.

## Supplementary Information

**Additional file 1.** CONSORT (CONsolidated Standards of Reporting Trials) 2010 Checklist. Recommended items to reporting of randomized controlled trials.

**Additional file 2: Supplementary file 1.** The process of designing and producing the BPMAP application.

**Additional file 3: Supplementary file 2.** More information about dairy, fruit and vegetable consumption, usability of the application and Portal analysis. There are three tables available in this file for each of them.

## Data Availability

The data sets used and analyzed during the current study are available from the corresponding author on reasonable request. The data cannot be presented, as the most important aspect of the intervention is the application and its online portal, which is in Persian, and is of no value to the non-Persian-speaking audience.

## Abbreviations

BPMAP: Blood Pressure Management Application
PRECEDE: Predisposing, Reinforcing, and Enabling Constructs in Educational Diagnosis and Evaluation
DASH: Dietary Approaches to Stop Hypertension
IPAQ: International Physical Activity Questionnaire
JNC: Joint National Committee
LDL: Low-density lipoprotein
HDL: High-density lipoprotein
BMI: Body mass index
MAP: Mean arterial pressure
