# Supplementary material for: The effect of the mobile “blood pressure management application” on hypertension self-management enhancement: a randomized controlled trial
Source: Trials. 2021 Jun 24;22:413. doi: 10.1186/s13063-021-05270-0 (PMC8223338; doi:10.1186/s13063-021-05270-0)
Supplement: Supplementary file 2 — Additional file 2: Supplementary file 1. The process of designing and producing the BPMAP application. [file 13063_2021_5270_MOESM2_ESM.docx]

**Supplementary file 1-** The process of designing and producing the BPMAP application and interface of app

The study was arranged in five steps (fig 1). 1-Defining the conceptual framework, bearing in mind the system’s properties and development of appropriate content, 2-Approving the conceptual framework and system of delivery, 3-Arrangement and adjustment of the conceptual framework and content (tailoring), bearing in mind the users’ characteristics, 4-Performance assessment, and, 5-Data collection and analysis. During the first to 4^th^ step, the software has been developed and debugged. In the 5^th^ step, a clinical trial was conducted through the controlled randomized clinical trial.

The interface of the application and some of the pages (Persian)


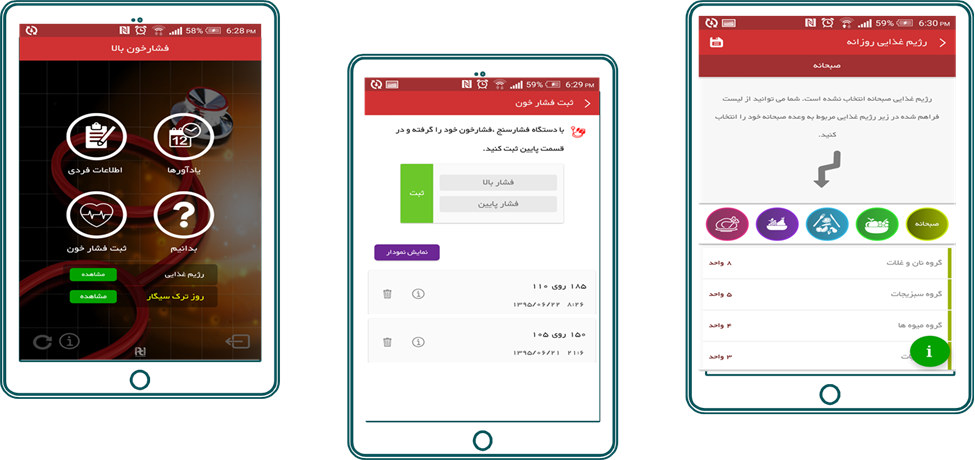


a b c

Smartphone application: a- the home screen of BPMAP, b- The record of BP measurement c- Writing a dash diet page
