# Supplementary material for: The effect of the mobile “blood pressure management application” on hypertension self-management enhancement: a randomized controlled trial
Source: Trials. 2021 Jun 24;22:413. doi: 10.1186/s13063-021-05270-0 (PMC8223338; doi:10.1186/s13063-021-05270-0)
Supplement: Supplementary file 3 — Additional file 3: Supplementary file 2. More information about dairy, fruit and vegetable consumption, usability of the application and Portal analysis. There are three tables available in this file for each of them. [file 13063_2021_5270_MOESM3_ESM.docx]

**Supplementary file 2-** More information about dairy, fruits and vegetables consumption, usability of application and Portal analysis.

Table 1- Portal analysis of users of BPMAP application

| Main parts | Items | | **N (%)** |
| --- | --- | --- | --- |
| Information about hypertension | Visit |  | **58(98.3)** |
|  | Type of information | text | **34(57.6)** |
|  |  | Audio | **8 (13.5)** |
|  |  | Both of them | **15 (25.4)** |
|  | Number of visits | once | **41 (69.4)** |
|  |  | Twice | **13(22.0)** |
|  |  | Three and more | **3 (5.0)** |
| Complete individual information |  |  | **59(100)** |
| Enter the nutrition section |  |  | **56 (94.9)** |
| Register at least one diet list |  |  | **33 (50.9)** |
| Enter the overweight section |  |  | **(4/72) 42** |
| The tendency to lose weight |  |  | **(8/42) 18** |
| Set reminders | types | visit | **(9/56) 33** |
|  |  | drug | **30(50.8)** |
|  |  | BP measurement | **18(30.5)** |
|  |  | Para clinical | **(7/1) 1** |
| Quit smoking | Decision | I can not | **(4/3) 2** |
|  |  | I will quit | **(4/3) 2** |
| Record HBPM* more than 25 times |  |  | **50(84.7)** |

* Home Blood Pressure Measurements

Table 2- The changes in dairy, fruit and vegetable consumption in both groups over time

|  | | **Baseline assessment** | | | | **8^th^ week** | | | | **24^th^ week** | | | |
| --- | --- | --- | --- | --- | --- | --- | --- | --- | --- | --- | --- | --- | --- |
|  | | Less than one a day | One a day | Two a day | Three or more a day | Less than one a day | One a day | Two a day | Three or more a day | Less than one a day | One a day | Two a day | Three or more a day |
| **Dairy** | Intervention group (N/%) | 26  (8/44) | 25  (1/43) | 5  (6/8) | 2  (4/3) | 1  (7/1) | 9  (5/15) | 48  (8/82) | 0 | 1  (7/1) | 10  (2/17) | 47  (0/81) | 0 |
|  | Control group (N/%) | 21  (0/35) | 32  (3/53) | 6  (0/10) | 1  (7/1) | 13  (7/21) | 37  (7/61) | 10  (7/16) | 0 | 18  (0/30) | 37  (7/61) | 5  (3/8) | 0 |
| **fruits** | Intervention group (N/%) | 16  (6/27) | 13  (4/22) | 19  (8/32) | 10  (2/17) | 0 | 2  (4/3) | 24  (4/41) | 32  (2/55) | 1  (7/1) | 23  (7/39) | 34  (6/58) | 0 |
|  | Control group (N/%) | 6  (0/10) | 21  (0/35) | 19  (7/31) | 14  (3/23) | 5  (3/8) | 27  (0/45) | 26  (3/43) | 2  (3/3) | 6  (0/10) | 28  (7/46) | 26  (3/43) | 0 |
|  | | Less than one a day | One or two a day | More than two a day |  | Less than one a day | One or two a day | More than two a day |  | Less than one a day | One or two a day | More than two a day |  |
| **vegetables** | Intervention group (N/%) | 31  (4/53) | 26  (8/44) | 1  (7/1) | - | 0 | 43  (1/74) | 15  (9/25) | - | 1  (7/1) | 46  (3/79) | 11  (0/19) | - |
|  | Control group (N/%) | 28  (7/46) | 26  (3/43) | 6  (0/10) | - | 35  (3/58) | 25  (7/41) | 0 | - | 30  (0/50) | 29  (3/48) | 1  (7/1) | - |

Table 3- Evaluation of usability of BPMAP application by users.

| **Mean(SD)** | **Description of characteristics**  5-point Likert scale (1=does not apply at all; 5=does fully apply) | **Sub-criteria** | Main criteria |
| --- | --- | --- | --- |
| **(5/0) 4/4** | Avoidance of foreign language and technical terms | **Use of understandable semantics** | Comprehensibility |
| **(5/0) 5/4** | Use of generally intelligible symbols and terms |  |  |
| **(6/0) 9/3** | If necessary, provision of additional explanations |  |  |
| **(5/0) 3/4** | Self-explanatory images and depictions, understandable without further support and explanations | **Simple comprehensibility and interpretability of displayed images and depictions** |  |
| **(5/0) 7/4** | Easily understandable and internally consistent menu structures | **Simple, self-explanatory menu structures** |  |
| **(5/0) 0/4** | Avoidance of strong hierarchical menu structures and too many functionalities |  |  |
| **(4/0) 8/4** | Clear, distinguishable colors for images and depictions or choice of color-neutral depictions | **Sufficient color contrast** | Presentation (Image and Text) |
| **(5/0) 6/4** | Avoidance of too glaring colors |  |  |
| **(5/0) 3/4** | Sufficient size of the screen , as well as input and output, fields | **Large size of operating elements** |  |
| **(5/0) 5/4** | Ability to adapt the size of operating elements and displayed images according to individual needs, capabilities, and preferences | **Ability to adapt the size of operating elements and displayed images** |  |
| **(5/0) 5/4** | Instant response to entered data, including easily understandable error messages in case of erroneous data input | **Instant and easily understandable feedback** | Usability |
| **(5/0) 6/4** | Ability to use the application without prior knowledge | **Intuitive usability** |  |
| **(5/0) 7/4** | Ease of learning |  |  |
| **(6/0) 1/4** | Fast achievement of the first feeling of success |  |  |
| **(5/0) 3/4** | Simple distinction between click-sensitive and non-click-sensitive areas, also without prior knowledge of the features of the touch screen technology | **Simple recognition of click-sensitive areas** |  |
| **(5/0) 5/4** | Reducing the probability of erroneous data input by limiting choice to meaningful values | **High fault tolerance/efficient fault management** | General characteristics |
| **(4/0) 8/4** | Efficient proofreading mode and/or helpful user feedback, for example, in case of erroneous data input |  |  |
| **(0/0) 2** | Avoidance of registration at online platforms (but partly contrary to data protection regulations) | **Password-protected services** |  |
